# Sequential immunization induces strong and broad immunity against all four dengue virus serotypes

**DOI:** 10.1038/s41541-020-00216-0

**Published:** 2020-07-24

**Authors:** Jue Hou, Shubham Shrivastava, Hooi Linn Loo, Lan Hiong Wong, Eng Eong Ooi, Jianzhu Chen

**Affiliations:** 1 Interdisciplinary Research Group in Infectious Diseases, Singapore-MIT Alliance for Research and Technology (SMART), Singapore, Singapore; 2 Emerging Infectious Diseases Program, Duke-NUS Graduate Medical School, Singapore, Singapore; 3 Koch Institute for Integrative Cancer Research and Departments of Biology, Massachusetts Institute of Technology, Cambridge, MA USA

**Keywords:** DNA vaccines, Dengue virus

## Abstract

A major challenge in dengue vaccine development is the need to induce immunity against four dengue (DENV) serotypes. Dengvaxia®, the only licensed dengue vaccine, consists of four variant dengue antigens, one for each serotype. Three doses of immunization with the tetravalent vaccine induced only suboptimal protection against DENV1 and DENV2. Furthermore, vaccination paradoxically and adversely primes dengue naïve subjects to more severe dengue. Here, we have tested whether sequential immunization induces stronger and broader immunity against four DENV serotypes than tetravalent-formulated immunization. Mice were immunized with four DNA plasmids, each encoding the pre-membrane and envelope from one DENV serotype, either sequentially or simultaneously. The sequential immunization induced significantly higher levels of interferon (IFN)γ- or tumor necrosis factor (TNF)α-expressing CD4^+^ and CD8^+^ T cells to both serotype-specific and conserved epitopes than tetravalent immunization. Moreover, sequential immunization induced higher levels of neutralizing antibodies to all four DENV serotypes than tetravalent vaccination. Consistently, sequential immunization resulted in more diversified immunoglobulin repertoire, including increased complementarity determining region 3 (CDR3) length and more robust germinal center reactions. These results show that sequential immunization offers a simple approach to potentially overcome the current challenges encountered with tetravalent-formulated dengue vaccines.

## Introduction

Dengue is a mosquito-borne viral disease with an estimated 100 million symptomatic infections every year. The etiological agent, dengue virus (DENV), consists of four antigenically distinct serotypes. Adaptive immunity elicited following infection with one DENV serotype does not confer lasting protection against subsequent infection by the other three heterotypic DENVs. At the amino acid sequence level, DENV1–4 share up to 70% sequence homology and therefore many conserved immunogenic epitopes. However, each DENV serotype also possesses unique linear and quaternary antigenic epitopes—antibodies that target such epitopes appear to be important for long-term serotype-specific immunity^1–4^. As cross-reactive antibodies mostly bind without neutralizing heterotypic DENVs, the resultant antibody-virus complexes enhance infection by enabling virus entry into phagocytic cells via Fc-gamma receptors (FcγR). Consequently, antibody-dependent enhancement (ADE) of DENV infection is associated with increased risk of severe dengue^5^.

Control of dengue requires a vaccine that induces effective protection against all four DENV serotypes while minimizing the risk of ADE. Currently, Dengvaxia® is the only licensed dengue vaccine. Dengvaxia® is a recombinant, live-attenuated, tetravalent dengue vaccine constructed by replacing the pre-membrane (prM) and envelope (E) genes of the 17D strain of the yellow fever vaccine with those from the four DENV serotypes. The tetravalent vaccine is administered in three doses at 6-month intervals. Unfortunately, the vaccine has limited overall efficacy (~60%) against acute dengue, with only 50% and 35–42% for DENV1 and DENV2, respectively^6–8^. Moreover, follow up studies have since shown that Dengvaxia® vaccination is associated with increased incidence of hospitalization and severe illness in vaccinees without prior dengue infection. For this reason, the World Health Organization (WHO) has recommended that Dengvaxia® be only used in those with serological evidence of prior dengue infection. The suboptimal efficacy and safety of Dengvaxia® underscore the need for developing new dengue vaccines and immunization strategies that stimulate balanced and long-lasting immunity against all four DENVs.

One approach is to induce immune responses against the conserved epitopes so as to achieve protection against all four serotypes of DENV without ADE. Indeed, studies have shown that although humoral response to the first antigen exposure shapes response to subsequent antigen exposure^9^, original antigenic sin^10^ can be overcome by the use of appropriate adjuvants or repeated immunization with the second antigen^11^. Recent studies, especially those in inducing broad neutralizing antibodies against HIV, have shown that sequential immunization with related immunogens could induce protective immunity towards the conserved and shared antigenic epitopes^12–15^. For example, sequential immunization was shown to induce neutralizing antibody responses not only to homologous but heterologous viruses^16–21^. In particular, Wang et al.^22^ reported B-cell responses and antibody maturation under different immunization strategies through computational modeling^23^. Their modeling of germinal center (GC) reaction suggests that simultaneous immunization with variant antigens of highly mutable pathogens leads to conflicts in selection that impair antibody maturation. In contrast, sequential immunization promotes B cells to interact on the footprint of the conserved epitopes that is induced by previous immunization and educate the B cells to achieve cross-reactive immune responses. They validated their model by successful induction of broad and high-affinity antibody responses through sequential immunization with four HIV immunogen variants. This promising finding suggests that sequential immunization strategy could be used to induce balanced immune responses to highly homologous dengue epitopes to achieve effective protection against four DENV serotypes.

In this study, we have compared the strength and breadth of both T- and B-cell immune responses to four dengue serotype DNA vaccines that are given either sequentially or simultaneously for four times. Our results show that sequential vaccination induces stronger and broader cellular and humoral immune responses than tetravalent immunization. We further studied immunoglobulin (Ig) diversification in antigen-specific B cells after each immunization to understand the evolution dynamics in different immunization approaches. Our findings have significant implication on how to improve the efficacy and safety of dengue vaccine through sequential immunization and vaccine development for highly variant pathogens in general.

## Results

### Sequential immunization induces stronger and broader T-cell responses than tetravalent immunization

**Fig. 1:**
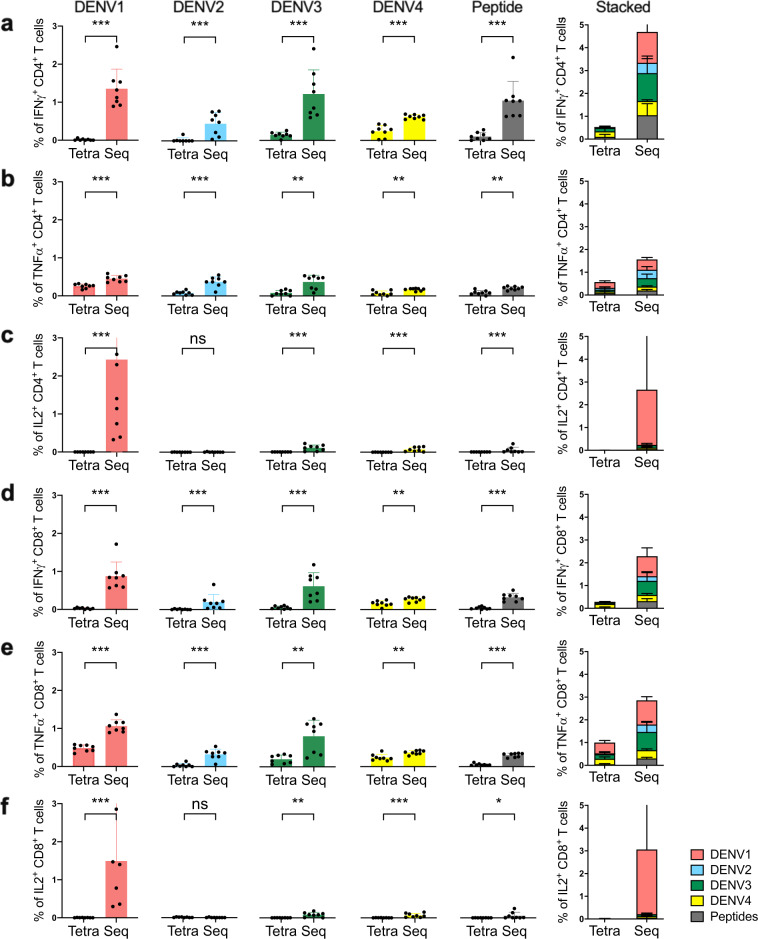
Sequential immunization (Seq) induces stronger and broader antigen-specific T-cell responses than tetravalent immunization (Tetra). Mice were sacrificed 2 weeks after the last immunization. Splenocytes were stimulated with each of the four DENV (DENV1/2402DK1, DENV2/3295DK1, DENV3/863DK1 and DENV4/2240DK1, at M.O.I = 1) or consensus envelope and capsid peptide pool (at a final concentration 5 µg/ml per peptide) or medium as control for 6 h at 37 °C in the presence of BFA. Cells were surface stained for CD3, CD4 and CD8, and intracellularly stained for IFNγ, TNFα, and IL2. The plots show the percentages of IFNγ, TNFα or IL2 expressing CD4^+^ (**a**–**c**) and CD8^+^ (**d**–**f**) T cells. The bar plots show the mean percentages (±SD) of cytokine-expressing CD4^+^ and CD8^+^ T cells to each DENV or peptides (left), and the stacked plots show the combined percentages (±SD) of cytokine-expressing CD4^+^ and CD8^+^ T cells (right). The colors indicate antigens used for stimulation. The *p*-values (ns: not significant (*p* > 0.05), *p < 0.05, **p < 0.01, ***p < 0.001, ****p < 0.0001) between the indicated comparisons were calculated by Mann–Whitney test (*n* = 8 mice per group).

We compared T-cell immune responses between sequential immunization and tetravalent immunization (Table 1). Two weeks following the last sequential or tetravalent immunization, splenocytes were harvested and stimulated with a mixture of consensus DENV peptides or each serotype of DENV and analyzed for cytokine expression by CD4^+^ and CD8^+^ T cells. The gating strategy was shown in the Supplementary Fig. 1. Overall, the total percentages of CD4^+^ T cells that were IFNγ^+^ (Fig. 1a) or TNFα^+^ (Fig. 1b) from tetravalent immunized mice were ~0.5% following stimulation with all antigens, whereas the total percentages of CD4^+^ T cells that were IFNγ^+^ or TNFα^+^ from sequentially immunized mice were 4.5% and 1.5% (~3–9-fold increase), respectively. Similarly, the total percentages of CD8^+^ T cells that were IFNγ^+^ or TNFα^+^ from sequentially immunized mice were 3 to 10 times higher than those that received tetravalent immunization (~2.5% vs. 0.25–1%, Fig. 1d, e). The sequential immunization induced up to 10 times higher immune responses against conserved peptides in both CD4^+^ (Fig. 1a–c) and CD8^+^ (Fig. 1d–f) T cells than the corresponding response following the tetravalent immunization. Following tetravalent immunization, the highest percentages of CD4^+^ and CD8^+^ T cells expressed TNFα in response to DENV1 re-stimulation, but these were still significantly lower than animals that were sequentially immunized (~0.25% vs. ~0.45% in CD4^+^ T cells, *p* = 0.0002 and ~0.48% vs. ~1.1% in CD8^+^ T cells, *p* = 0.0002).

**Table 1:** Comparison of tetravalent and sequential immunization scheme.

Group	Immunization	1st shot	2nd shot	3rd shot	4th shot
1	Tetravalent	Tetravalent	Tetravalent	Tetravalent	Tetravalent
2	Sequential	Den1	Den2	Den3	Den4

Serotype-specific T-cell responses were significantly higher following sequential immunization than tetravalent immunization. For instance, the percentage of IFNγ -expressing CD4^+^ (Fig. 1a) or CD8^+^ (Fig. 1d) T cells were much higher against DENV3 (0.15% in CD4^+^, 0.06% in CD8^+^) and DENV4 (0.24% in CD4^+^, 0.16% in CD8^+^) than DENV1 (0.01% in CD4^+^, 0.03% in CD8^+^) and DENV2 (0.02% in CD4^+^, 0.01% in CD8^+^) among tetravalent immunized mice, a difference of 2–20-fold between the strongest and the weakest responses. In contrast, the percentages of IFNγ-producing CD4^+^ or CD8^+^ T cells were not significantly different among the DENV serotypes following sequential immunization: 1.35%, 0.45%, 1.22% and 0.62% in CD4^+^ T cells and 0.88%, 0.20%, 0.62% and 0.27% in CD8^+^ T cells specific for DENV1, DENV2, DENV3, and DENV4, respectively. The serotype-specific CD4^+^ and CD8^+^ T-cell responses were lowest to DENV2 as compared to the other three DENV serotypes following the sequential immunization. Notably, most IL2 expressing CD4^+^ or CD8^+^ T cells were stimulated by DENV1, the first immunizing antigen used during sequential immunization (Fig. 1c, f), whereas no or very little IL2 expressing CD4^+^ or CD8^+^ T cells were stimulated by DENV2–4. These results show that sequential immunization induced stronger T-cell responses than tetravalent immunization, as well as broadened T-cell responses to all four serotypes, especially DENV1 and DENV2.

### Sequential immunization enhances and broadens the neutralizing antibody responses

**Fig. 2:**
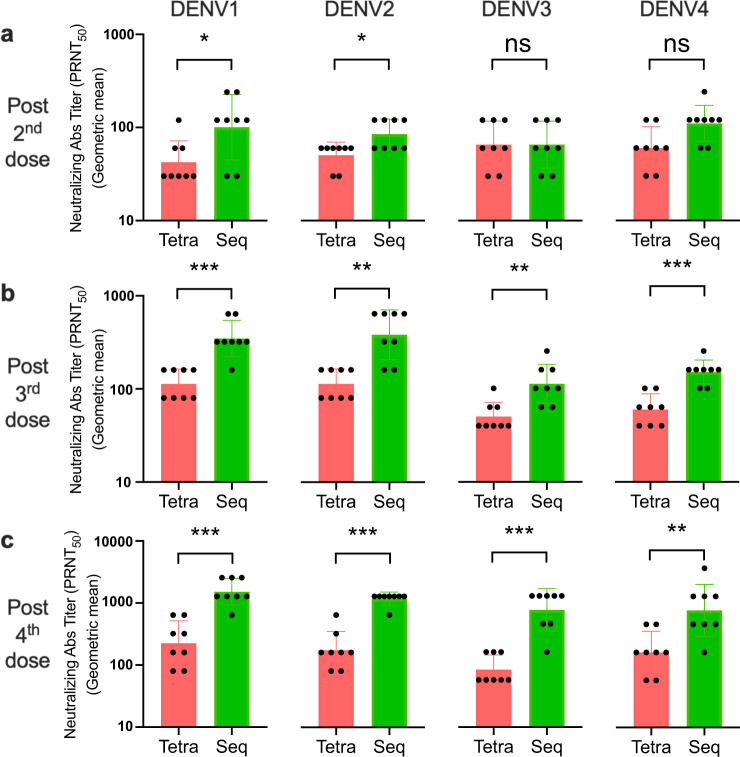
Sequential immunization enhances and broadens neutralization antibody responses. The neutralization antibody titers 2 weeks after the 2nd (**a**), 3rd (**b**), and 4th (**c**) immunization were measured by plaque reduction neutralization test (PRNT) assay. Four serotypes of dengue virus were separately incubated with serially diluted sera to measure the neutralization capability of reactive antibodies. The serotype-specific neutralizing antibodies were determined by 50% plaque reduction compared to the virus control wells. The data shown are geometric mean titers ± geometric SD in the bar plot. The *p-*values (ns: not significant (*p* > 0.05), *p < 0.05, **p < 0.01, ***p < 0.001, ****p < 0.0001) between the indicated comparisons were calculated by Mann–Whitney test (*n* = 8 mice per group).

We compared the titers of serotype-specific neutralizing antibody (nAb) in serum samples of immunized mice after tetravalent and sequential immunization. As expected, increase in nAb titers after each immunization in both groups were observed. The overall nAb titers were significantly higher in sequential immunized mice than in tetravalent immunized mice after the 3rd and the 4th immunization (Fig. 2). For instance, anti-DENV2 titers elevated 2.3 times between 2nd and 3rd tetravalent immunization (geometric mean value 50 vs. 113), whereas the antibody titers increased ~4.5 times between 2nd and 3rd sequential immunization (geometric mean value 85 vs. 380). By the 4th tetravalent immunization, the anti-DENV2 nAb titer was 175 (geometric mean value), which was lower than those against the other DENV serotypes (geometric mean value of 226, 414, and 640 for anti-DENV1, DENV3, and DENV4 nAb titers, respectively). In contrast, by the 4th sequential immunization, the titer of nAb against DENV2 was 1174 (geometric mean value), which was still lower but more comparable to those against the other serotypes (geometric mean value of 1522, 1810, and 1810 for anti-DENV1, DENV3, and DENV4 nAb titers, respectively) (Fig. 2c). Thus, sequential immunization induces stronger neutralizing antibody responses than tetravalent immunization does.

### Sequential immunization improves the antigen-specific memory B-cell responses

**Fig. 3:**
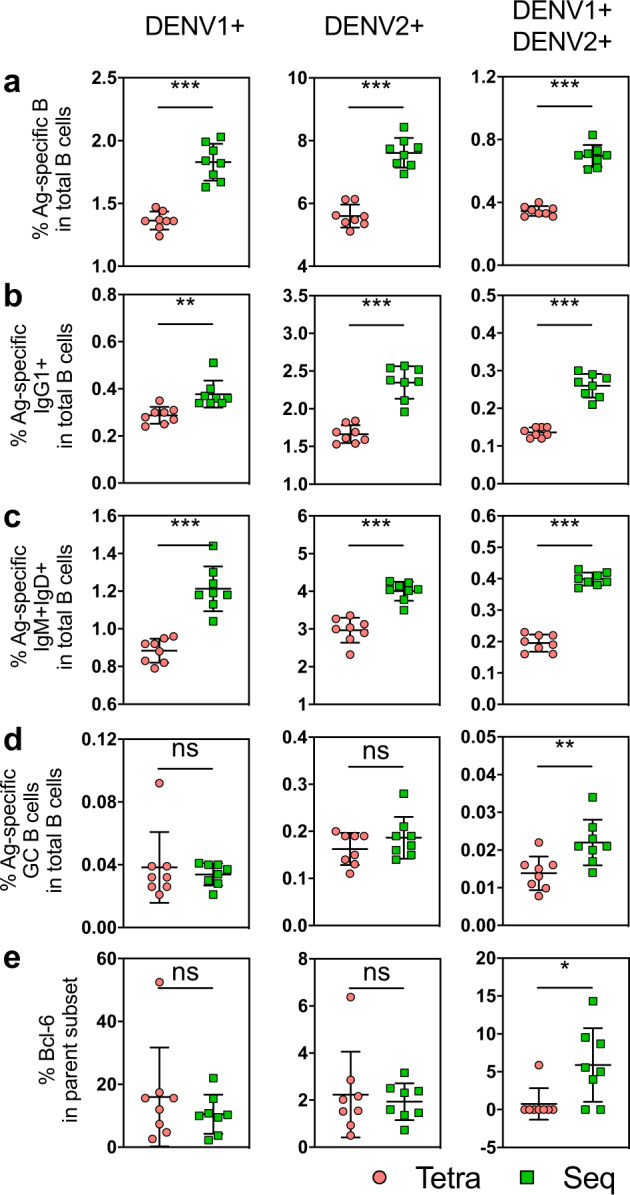
Comparison of antigen-specific B-cell responses in tetravalent and sequential immunized mice. Two weeks after last immunization, splenocytes were stained with Alexa conjugated DENV1 and DENV2 E proteins and appropriate antibodies (see list in “Methods”). The antigen-specific DENV1^+^ (left panel), DENV2^+^ (middle panel) and DENV1^+^DENV2^+^ (right panel) B cells (**a**), IgG1^+^ B cells (**b**), IgM^+^IgD^+^ B cells (**c**), and germinal center B cells (**d**), and their corresponding Bcl6 expression (**e**) were assessed. Each dot represents one mouse and mean ± SD are shown. The *p*-values (ns: not significant (*p* > 0.05), *p < 0.05, **p < 0.01, ***p < 0.001, ****p < 0.0001) were calculated by Mann–Whitney test (*n* = 8 mice per group).

To quantify antigen-specific B-cell responses following sequential and tetravalent immunizations, we utilized DENV1/E-AF647 and DENV2/E-AF548 to stain for DENV1 and DENV2-specific B cells in the spleen 2 weeks after the last immunization (Supplementary Fig. 2). In comparison to tetravalent immunization, sequential immunization induced significantly higher levels of DENV1^+^ or DENV2^+^ single-positive, and cross-reactive (DENV1^+^ and DENV2^+^ double positive) B cells (Fig. 3a). Consistently, sequential immunization increased the levels of DENV1^+^ (left panel), DENV2^+^ (middle panel), and DENV1^+^DENV2^+^ (right panel) specific IgG1^+^ (Fig. 3b) and IgM^+^IgD^+^ (Fig. 3c) B cells as compared to tetravalent immunization. Sequential immunization also significantly elevated the levels of DENV1^+^DENV2^+^ germinal center (GC) B cells, but not DENV1^+^ or DENV2^+^ GC B cells (Fig. 3d), which was consistent with increased expression of the transcriptional factor BCL6 (Fig. 3e), critical for GC formation and maintenance^24^.

**Fig. 4:**
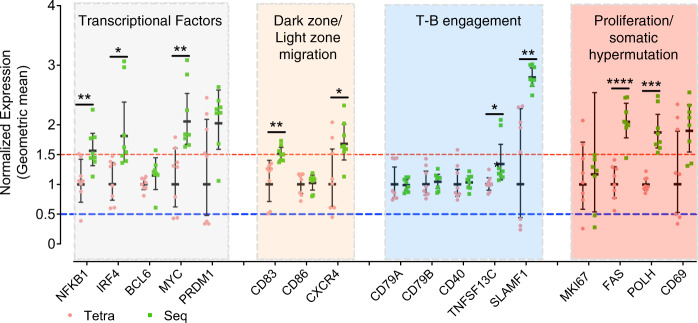
Antigen-specific germinal center B-cell profiling by Taqman qPCR. Single-DENV1^+^DENV2^+^ germinal center B cell was sorted by flow cytometry into 96-well plate. The cells were lyzed and reverse transcribed into cDNA. The levels of the selected transcripts were quantified by Taqman qPCR. The ACTB was used as a reference gene. The results were calculated by delta Ct method and normalized to those of tetravalent immunization. The individual dot represents a single cell and the geometric mean value ± 95% CI are shown. The geometric mean values are considered significant if the differences are 1.5-fold between the sequential group (green) and tetravalent group (red). The asterisk indicates a significant difference between the indicted groups (*t*-test, *p < 0.05, **p < 0.01, ***p < 0.001, ****p < 0.0001).

To better understand the molecular basis underlying the observed difference in GC reactions between tetravalent and sequential immunizations, we sorted single antigen-specific GC B cell (DENV1^+^DENV2^+^CD38^-^GL7^+^B220^+^) and performed quantitative reverse transcription PCR (qRT-PCR) on a panel of well-characterized genes that are involved in germinal center reaction, including transcription factor (NF-κB, IRF4, BCL6, MYC, PRDM1), cell–cell interaction and migration (CXCR4, CD83, CD86, CD79, CD40, TNFSF13C, SLAMF1), proliferation and hypermutation (MKi67, FAS, Pol ƞ, CD69)^25,26^. As shown in Fig. 4, the sequential immunization enhanced the expression of transcriptional factors NF-κB, IRF4, and MYC involved in GC reaction; CD83 and CXCR4 involved in transition between the dark and light zone in the GC; TNFSF13C and SLAMF1 involved in the interactions between Tfh and B cells; and the DNA polymerase Pol ƞ and FAS involved in DNA recombination and cell proliferation, in comparison with tetravalent immunization. Overall, sequential immunization significantly enhances antigen-specific B-cell responses by facilitating GC reactions.

### Sequential immunization broadens the immunoglobulin diversity

**Fig. 5:**
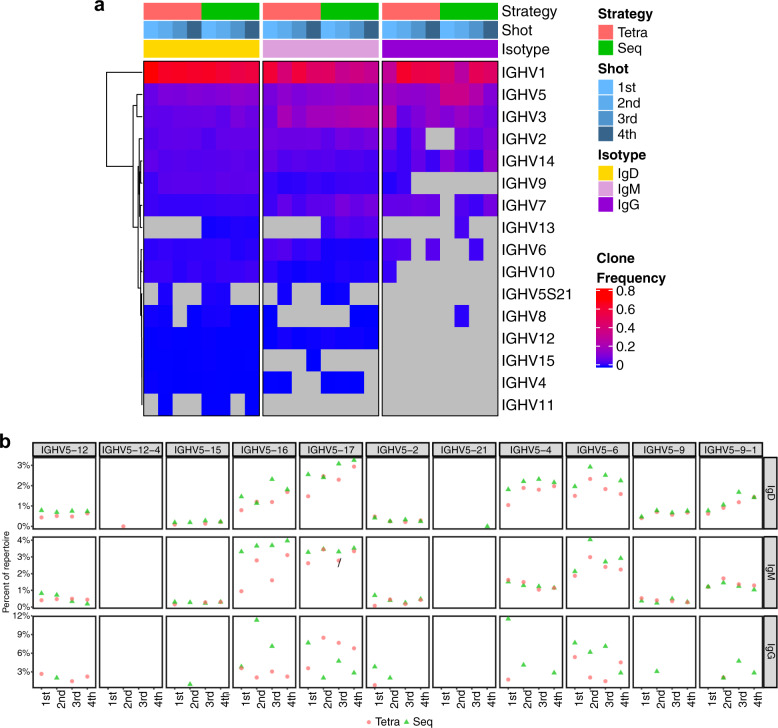
Comparison of VH gene usages by two strategies at different dosage of immunization. **a** Heatmap shows the V_H_ family usages by clone frequencies with red to blue to gray corresponding to from high to low to absence. Shot indicates the order of immunization and isotype indicates IgD, IgM and IgG. **b** Comparison of IGHV5 family gene usage frequency between tetravalent and sequential immunizations. The Chi-square test was performed for statistical analysis.

To compare the diversity of Ig repertoires between tetravalent and sequential immunizations, we performed the Ig RNA sequencing on sorted DENV1^+^ and/or DENV2^+^ B cells after each immunization of two strategies (Table 2). Overall, the breadth of Ig heavy chain variable gene usage was not different between the two immunization strategies, but it decreased as DENV-specific B cells differentiated from IgM^+^/IgD^+^ to IgG^+^. The IgG^+^ B cells predominantly used IGHV1, 2, 3, 5, 7, and 14, consistent with V_H_ gene usages in the acute and convalescent dengue patients^27^. Based on the clone frequencies, the IGHV1 was the dominant family used by DENV^+^ B cells regardless of Ig isotypes. IGHV5 family usage was elevated in DENV^+^ B cells that expressed either IgD or IgG (Fig. 5a). Both immunizations induced IGHV5 usage that was more prominent following sequential immunization (e.g., IGHV5-4, −6, −16, IGHV5–17 etc. detailed in Fig. 5b) but without statistical significance.

**Table 2:** The summary of RNA-seq results.

Group	Original sequences	Assembled sequences	Filtered sequences	IgBlast clones	Final repertoire
Tetra 1st	645,878	593,797	18,541	73,663	64,336
Seq 1st	808,887	743,239	25,139	99,715	89,704
Tetra 2nd	691,792	637,020	24,724	98,419	88,891
Seq 2nd	722,551	661,577	30,542	121,773	110,844
Tetra 3rd	663,385	613,046	28,481	113,354	103,124
Seq 3rd	750,832	692,652	50,863	202,772	183,156
Tetra 4th	769,545	695,547	37,098	148,002	132,753
Seq 4th	843,287	780,215	37,989	151,484	136,656

**Fig. 6:**
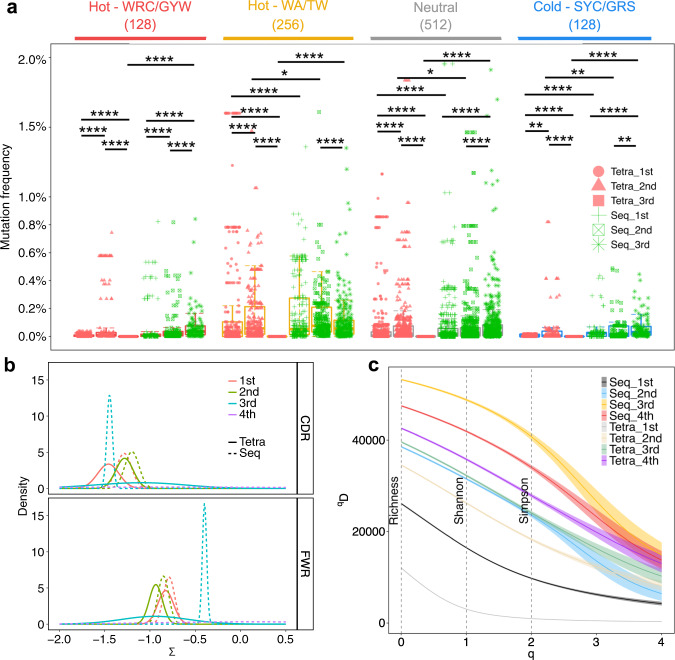
Comparison of the BCR diversity and somatic hypermutations between two immunization strategies. The bar plots for the levels of somatic hypermutation (SMH) in hot- and cold spots. SMH targeting profiles were analyzed for 1024 5-mer motifs from the 1st, 2nd, and 3rd dose of both immunization strategies. The 128 WRC/GYW hotspot motifs (red), 256 WA/TW hotspot motifs (orange), 512 neutral spots (gray), and 128 SYC/GRS cold spot motifs (blue) are shown. Each dot represents a 5-mer motif and each box covers the 25th–75th percentiles of the mutability rates of the 5-mer motifs in its corresponding groups, with the horizontal bar indicating the median. The *p*-values show the statistical significance by Wilcoxon test analysis for indicated groups (*p < 0.05, **p < 0.01, ***p < 0.001, ****p < 0.0001). **b** BASELINe method was used to calculate the posterior distribution of selection strengths (∑) for CDR (upper panel) and FWR (lower panel) after each immunization. The tetravalent and sequential immunizations are indicated by solid and dashed line, respectively. The numbers of immunization are indicated by different colors. **c** The clonal diversity analysis was performed by using the generalized Hill’s diversity index. The diversity index (^*q*^*D*) was calculated over a range of diversity orders (*q*) and plotted as a smooth curve. The ^*q*^*D* values depict the level of diversity for a given value of *q*. The lower ^*q*^*D* values represent lower diversity. Shaded area represents 95% percentiles. The Richness diversity index, which equates to *q* = 0, the Shannon diversity index, *q* = 1, and the Simpson diversity index, *q* = 2, were plotted as dashed vertical lines.

Replacement mutations were identified in complementarity determining regions (CDR) and framework regions (FWR) by comparing input sequences with the corresponding germline sequences. Tetravalent immunization generated higher mutation frequencies in IgD isotype at any given dose and in IgM isotype at first two doses than sequential immunization (Supplementary Fig. 3a). Moreover, tetravalent immunization also induced more mutations in FWR in IgG isotype at the 2nd and 4th doses (Supplementary Fig. 3b). Somatic hypermutation (SHM) was analyzed after each dose of both immunizations and was found at hot spots at the 1st and 2nd doses of both immunizations and continued to occur at the 3rd dose of sequential immunization but not at the 3rd or 4th dose of tetravalent immunization (Fig. 6a). Additionally, sequential immunization induced both higher WA/TW hotspot and SYC/GRS cold spot mutation frequencies than tetravalent immunization after each dose. By Bayesian estimation of antigen-driven selection in BCR Sequences (BASELINe) analysis, the negative selection pressures were intensive on CDR than FWR in both immunization strategies (Fig. 6b). Moreover, the tetravalent immunization increased negative selection strength after the 1st and 2nd doses than sequential immunization, whereas sequential strategy maintained the negative selection until the 3rd dose (Fig. 6b). Furthermore, for both immunization strategies, selection strengths in CDR at the 1st dose were higher than at the 2nd dose, but opposite in FWR. The 3rd dose of sequential immunization was able to elicit considerable selection strength in CDR, but comparatively limited selection in FWR region. With the outcomes of mutation and selection, Ig repertoires became more diverse following more rounds of immunization by both immunizations (Fig. 6c). However, the Ig diversities were lower after the 1st and 2nd doses of tetravalent immunization than the corresponding sequential immunization. The Ig diversification after the 3rd dose of tetravalent immunization was similar to the diversity after the 2nd dose of sequential immunization, followed by limited increase at the 4th dose. The Ig diversity reached the maximum at the 3rd dose of sequential immunization but decreased at the 4th dose, which was still more diverse than tetravalent immunization at the same dose. In terms of CDR3 characteristic, sequential immunization selected longer CDR3 with less hydrophobicity but more frequent acidic amino acid residues (Supplementary Fig. 4).

Altogether, these results show that sequential immunization progressively induces somatic hypermutations and selects for more diverse Ig repertoires to achieve stronger and broader neutralizing antibody responses than tetravalent immunization.

## Discussion

In this study, we compared the strength and breadth of both T- and B-cell immune responses to four dengue serotype DNA vaccines that were administrated either sequentially (one serotype per dose) or simultaneously (cocktail comprise of four serotypes) for four times. We measured CD4^+^ and CD8^+^ T cells from the spleen of immunized mice 2 weeks after the last immunization to determine expression of IFNγ, TNFα, and IL2 following stimulation with each of the DENV serotypes or consensus peptides from prM and E. Overall, percentages of cytokine-expressing CD4^+^ and CD8^+^ T cells were approximately three to ten times higher following sequential immunization than tetravalent immunization (Fig. 1), suggesting the induction of stronger T-cell immune responses by sequential immunization. Although CD4^+^ and CD8^+^ T-cells responses to DENV2 were the lowest compared to the other three serotypes following sequential immunization, they were significantly higher than those to DENV2 following tetravalent immunization. The percentages of CD4^+^ and CD8^+^ T cells that expressed IFNγ, TNFα and especially IL2 were generally the highest following stimulation with DENV1, the first serotype vaccine used in sequential immunization, as compared to those induced by other three serotypes. These results suggest that while the order of immunization in the sequential immunization may contribute to the strength of immune responses, the nature of antigen also plays an important role because DENV2 was the second serotype vaccine used in the sequential immunization.

Sequential immunization also induced stronger and more diverse neutralizing antibody responses. Overall, the neutralizing antibody titers were approximately two-, four-, and threefold higher after the second, third, and fourth dose of sequential immunization than the same number of tetravalent immunization, respectively (Fig. 2). The neutralizing antibody titers against each of the four DENV serotypes were similar after each dose of sequential immunization. In contrast, DENV2 neutralizing antibody titers were significantly lower than those against DENV1, 3, and 4 after the fourth tetravalent immunization although the titers were similar after the second and third tetravalent immunization. Most remarkably, after the first two doses of sequential immunization with DENV1 and DENV2 (Fig. 2a), the titer of neutralizing antibody against DENV3 and DENV4 were as high as those to DENV1 and DENV2, suggesting that neutralizing antibodies likely cross-recognize different DENV serotypes.

ADE has been a significant concern for dengue vaccine development. Multiple doses of sequential immunization could accentuate this problem, especially when compliance of vaccination is poor. Based on our data using DNA vaccines encoding prM and E, similar neutralizing Ab responses were induced against all four DENV serotypes after the second to fourth immunization (Fig. 2), suggesting that ADE may not be a significant issue. This could be because only prM and E were used as immunogens, whereas non-neutralizing antibodies against other viral proteins are also induced following natural DENV infection. Previous cohort studies^28,29^ have shown that the risk of individuals to develop severity dengue illness following a third or fourth DENV exposure was low. Several vaccine trials have shown that the sequentially inoculated mono- or bi-valent vaccine do not increase the risk for severe diseases^30,31^. Altogether with our data, it may be possible to induce protection against all four DENV serotypes with just two or at most three doses of sequential immunization.

Several reports have suggested that primary infection with DENV1 and DENV3 is more likely to be symptomatic than DENV2 and DENV4^32,33^; the latter two serotypes appear to cause more symptomatic secondary infection than the former serotypes^32,34^. These observations raise the possibility that DENV2 and DENV4 could take advantage of antibody-dependent infection pathway more effectively than DENV1 and DENV3. Despite being more likely to cause symptomatic infection, however, there is no evidence that immunity to DENV2/DENV4 is dependent on prior infection with DENV1/DENV3. Indeed, the highest efficacies from phase 3 trials of Sanofi Pasteur’s Dengvaxia®^8^ and Takeda Vaccines’ TAK003^35^ were DENV4 and DENV2, respectively, even following a single dose in vaccines without prior dengue infection^8,36^. Therefore, sequential immunization could benefit from priming with DENV2/DENV4 followed by secondary vaccination with DENV1/DENV3. We used DENV1 first and DENV2 second during the sequential immunization because protection against DENV1 and DENV2 was lower as compared to DENV3 and DENV4 following Dengvaxia® vaccination. In retrospect, we should have used DENV2 first during the sequential immunization.

Our sequence analysis of immunoglobulin genes in DENV-reactive B cells by RNA-seq shed lights on diversity, mutation and selection of Ig repertoires induced by sequential and tetravalent immunizations. Following both immunizations, more diverse IGVH gene usages were detected in DENV-reactive IgM^+^/IgD^+^ B cells than IgG^+^ B cells (Fig. 5), suggesting selection as DENV-reactive B cells switch to IgG isotype. Interestingly, higher mutation frequencies in the DENV-reactive IgD^+^ B cells were detected after each dose of tetravalent immunization than sequential immunization (Supplementary Fig. 3), suggesting the antigen complexity in the tetravalent vaccine. Nevertheless, sequential immunization boosted dramatically high levels of DENV1^+^, DENV2^+^, and double-positive IgM^+^/IgD^+^, and IgG^+^ B cells (Fig. 3).

Antibody affinity maturation proceeds in a stepwise fashion as B cells experience iterative cycles of mutations and selections in the GC^37,38^, which go through the selection in the light zone (LZ) and return to the dark zone (DZ) for further rounds of proliferation, mutation, and selection^39,40^. B-cell gene profiling suggested high GC reaction induced by sequential immunization (Fig. 4). Transcription factors such as NF-κB, IRF4, MYC and PRDM1 associated with GC response^41,42^ were higher in DENV-reactive B cells following sequential immunization than tetravalent immunization (Fig. 4). More critical molecules involved multiple steps in GC reaction, including B-cell migration between DZ and LZ (e.g., CD83 and CXCR4)^43,44^, T–B-cell interaction (SLAMF1)^45^, B-cell proliferation and somatic hypermutation (e.g., FAS, POLH and CD69)^25,46,47^ were upregulated following sequential immunization. Somatic hypermutation (SHM) is initiated by activation-induced cytidine deaminase (AID) and occurs prior to antigen affinity-driven selection^48,49^. AID preferentially targets specific motifs, or hot spots of SHM, such as WRC/GYW and WA/TW (where W = {A, T}, R = {G, A}, Y = {C, T}, and the mutated nucleotide is underlined), and DNA polymerase ƞ (Pol ƞ) is known to introduce the mutation in the WA/TW motif^50^. We show that the sequential immunization induced a higher level of Pol ƞ expression (Fig. 4) compared with tetravalent immunization, accompanied with higher mutation frequency in the WA/TW motif and lasted till the 3^rd^ dose of sequential immunization (Fig. 6a). Sequential immunization also led to mutations at the cold spot, suggesting potential diversification of Ig repertoire.

The principle of sequential immunization generally aligns with the reality for individuals living in dengue endemic areas, whose immune responses may become protective after multiple heterotypic exposures^30,51,52^. Consistently, the efficacy of Dengvaxia® was significantly lower in DENV-naive individuals (35% efficacy) than those who were DENV seropositive at baseline (74% efficacy)^8^, suggesting that the pre-existing DENV immunity may be beneficial for vaccination outcome. Infection with a single-DENV serotype elicits only short-term cross-protective immunity against other heterologous serotypes DENV^4,53^, but the duration of cross-protection remains controversial, which varies from weeks to years^52,54^. Our study shows that within the period of cross-protection following monovalent vaccine immunization, subsequent boost with heterologous monovalent vaccine could reinforce the heterotypic immunity to achieve protective immunity against to all four DENV serotypes.

## Methods

### DNA vaccines

The DNA fragments containing pre-membrane (prM) and envelope (E) gene, from Dengue 1/2402DK1 (GenBank: EU081230.1), Dengue 2/3295DK1 (GenBank: EU081177.1), Dengue 3/863DK1 (GenBank: EU081190.1), and Dengue 4/2270DK1 (GenBank: GQ398256.1) were synthesized using humanized codons and preceded by a consensus Kozak sequence at −6 nucleotides to maximize protein expression, and cloned into plasmid NTC7482 (Nature Technology)^55^ under the control of the human cytomegalovirus promoter and intron A followed by a bovine growth hormone polyadenylation signal. DNA plasmids were prepared by Endotoxin-free Giga Plasmid Kit (Qiagen) and adjusted to 4 mg/ml. All DNA plasmids were aliquot and maintained at −80 °C until use. Plasmid DNA vaccine candidates were transfected into 293T cells (ATCC® CRL-3216^™^) and expression of the envelop protein was confirmed by Western blotting.

### Mice and immunization regimens

C57BL/6J (B6) mice were used for all experiments. Mice were bred and housed at the Animal Facility, National University of Singapore (NUS). All procedures and care were approved by the NUS Research Ethics Committee under Protocol R13-6157. All ethical regulations regarding animal research were complied with.

Table 1 depicts the immunization schedule of tetravalent and sequential vaccination strategies. For tetravalent immunization, a total of 50 µg plasmid DNA of each serotype in 50 µl phosphate-buffered saline (PBS); (12.5 µl per serotype DNA was premixed) was used per injection. For sequential immunization, 50 µg DNA of each serotype in 50 µl PBS was used per injection. The same amount of DNA plasmid(s) was used at each shot of each regimen. Two groups of 8-week-old female B6 mice (eight mice per group) were immunized four times intramuscularly each with 50 µl DNA plasmids under general anesthesia using BD Insulin Syringe with Ultra-Fine needle (31G 1cc 5/16”). The intervals between each immunization were 2 weeks. Two weeks after the final dose, the mice were sacrificed for terminal analysis.

### Intracellular cytokine staining

Splenocytes from immunized mice was assessed for cytokine production by intracellular cytokine staining as described previously^56,57^. Briefly, 1 million splenocytes were stimulated with a peptide cocktail (1 prM and 2 E peptides, at a final concentration of 5 μg/ml for each peptide)^58^ or each serotype virus (DENV1/2402DK1, DENV2/3295DK1, DENV3/863DK1 and DENV4/2240DK1, at a final M.O.I of 1) for 6 h at 37 °C in the presence of Brefeldin A (Golgiplug, BD Biosciences). These DENVs were all low-passage clinical isolates obtained from blood samples of acute dengue fever patients in Singapore^59^. Cells were surface stained with anti-CD3, anti-CD4, anti-CD8, anti-F4/80, anti-CD11c, anti-Ly6G, and anti-NK1.1 antibodies and LIVE/DEAD stain FSV780 (BD Biosciences) on ice for 30 min. For intracellular staining, cells were fixed and permeabilized with Fix/Perm buffer (BD Biosciences) for 30 min at 4 °C in the dark, incubated with anti-IL2, anti-TNFα, and anti-IFNγ monoclonal antibodies, followed by washing. A complete information of antibodies was presented in Supplementary Table 1. Cells were analyzed on LSRII flow cytometer (BD Biosciences) and data processed using FlowJo version 10.6.0 (Tree Star). The final readout of a given stimulation was calculated by subtracting mock control value from experimental data (DENV1–4 or peptide stimulation) for each sample. The results were considered as zero if the subtracted values were negative.

### Dengue plaque reduction neutralization test (PRNT)

Neutralizing antibody titer (nAb) was determined by PRNT as previously described^57,60,61^. Briefly, mouse sera were inactivated at 56 °C for 30 min and serially diluted with RPMI-1640 supplemented with 2% fetal bovine serum (FBS). Diluted sera were mixed with equal volume of DENV1/2402DK1, DENV2/3295DK1, DENV3/863DK1 or DENV4/2240DK1 (30–50 PFU/well) and incubated at 37 °C for 1 h. Virus-serum mixture was transferred onto BHK21 monolayer and allowed to absorb for 1 h at 37 °C. Cells were overlaid with 1% CMC with 2% FBS, antibiotics and NaHCO_3_ and incubated for 6 to 7 days at 37 °C in 5% CO_2_ incubator. After incubation, overlay media were removed and the cells were fixed in 7.5% formalin for 1 h, followed by washing with running tap water to remove residual CMC. 1% crystal violet solution was added onto the plates and stained for 1 h. The plates were washed in running water and air dried. The highest serum dilution that resulted in 50% or more plaques reduction compared to the virus control wells was considered as the neutralizing endpoint titer (PRNT_50_).

### B-cell assays

To measure the antigen-specific B-cell responses, we used protein labeling kits (Thermo Fisher Scientific) to conjugate Alexa Flour 647 (AF647) and Alexa Flour 548 (AF548) to extracellular domain of DENV1 (DENV1/VN/BID-V949/2007) and DENV2 (DENV2/GWL39 IND-01) E proteins (~50 kDa, CTK Biotech), respectively. One million splenocytes were incubated with DENV1/E-AF647 and DENV2/E-AF548 probes on ice for 30 min in the dark. The cells were then surface stained with conjugated anti-CD90.2, anti-F4/80, anti-CD11c, anti-CD4, anti-CD8, anti-Ly6G, anti-NK1.1, anti-IgM, anti-IgD, anti-GL7, anti-CD45R, anti-CD38 antibodies (Supplementary Table 1), and LIVE/DEAD stain FSV780 (BD Biosciences) on ice for 30 min. For the intracellular staining, cells were fixed and permeabilized with Phosflow Lyse/Fix buffer (BD Biosciences) for 10 min at 37 °C in the dark and subsequently incubated with Phosflow Perm/Wash buffer for 30 min at room temperature. Then, cells were stained with anti-Ig _(H+L)_ and anti-Bcl6 antibodies for 30 min at 4 °C in the dark. Cells were analyzed on an X20 flow cytometer (BD Biosciences) and data processed using FlowJo version 10.6.0 (Tree Star).

### Antigen-specific germinal center B-cell gene profiling by qPCR

Single-DENV1^+^DENV2^+^-specific germinal center (GC) B cell (B220^+^CD38^−^GL7^+^) was individually sorted by flow cytometry and analyzed the level of the selected transcripts. Briefly, 1 million splenocytes were stained as above. Single-GC B cell was sorted into 96-well PCR plate containing 20 µl RT-Pre-Amplification Master Mix (10 µl CellsDirect 2×Reaction Mix; 0.5 µl SuperScript III RT/Platinum Taq; 1 µl pooled outside gene-expression primers; 8.5 µl DEPC-water) (see Supplementary Table 2 for primers list^25^). The reverse transcription was performed at 50 °C for 15 min, followed by 95 °C for 2 min, then 30 cycles of 95 °C for 15 s, 60 °C for 4 min. The complementary DNA (cDNA) was processed for analysis of gene expression by using TaqMan Universal PCR Master Mix (see Supplementary Table 3 for TaqMan probes list^25^) with reaction condition of 50 °C for 2 min, followed by 95 °C for 10 min, then 40 cycles of 95 °C for 15 s, 60 °C for 1 min. The relative expression was calculated by the cycling threshold (Ct) values from individual genes according to the 2^-∆∆Ct^ method as reported previously^62,63^. Briefly, the average Ct of all samples in the control group (i.e., tetravalent immunization) for each gene is determined. The relative difference (ΔCt) between the average Ct for the control group and sequential immunization group for each gene is calculated. Then, the relative expression for each gene is normalized to the transcript level of the housekeeping gene ACTB followed by log transformation. The average normalized and log-transformed expression for each biological group is then calculated using the geometric mean (ΔΔCt). The standard deviation (SD), standard error of the mean (SEM), and 95% confidence interval of each group are then calculated from the log-transformed normalized expression.

### Antigen-specific B cells sorting and immunoglobulin repertoire sequencing by RNA-seq

The pan-B cells from immunized mice were pre-enriched by EasySep Mouse B-Cell Isolation Kit (Stemcell). 100–150 million splenocytes were incubated with DENV1/E-AF647 and DENV2/E-AF548 on ice for 30 min in the dark, and then surface stained with fluorescence-conjugated antibodies as above. After staining, B cells were washed and re-suspended in staining buffer (PBS with 2% FBS). Total 10,000 DENV-specific B cells (either DENV1^+^ or DENV2^+^ or DENV1^+^DENV2^+^) were sorted into FACS tubes with staining buffer supplemented with RNase Inhibitor cocktail using a FACS Aria II cell sorter (BD Biosciences). RNA was extracted from sorted cells by RNeasy Micro Kit (Qiagen) and the quality of RNA was assessed by Nanodrop and quantified by Qubit Fluorometer. RNA was reverse transcribed into cDNA by 10×SMARTScribe Reverse Transcriptase (Clontech). The cDNA was purified using a MinElute PCR Purification Kit (Qiagen). The Ig heavy (H) genes were amplified by two-round nested PCR using published primers (included µ chain, γ chain, and δ chain) (Supplementary Table 4) and PCR conditions^64–66^. Briefly, the first round of PCR was performed at 94 °C for 15 min followed by 50 cycles of 94 °C for 30 s, 56 °C for 30 s, 72 °C for 55 s, and final incubation at 72 °C for 10 min. Semi-nested or nested second round PCR was performed with 3.5 μl of unpurified first round PCR product at 94 °C for 15 min followed by 50 cycles of 94 °C for 30 s, 60 °C for 30 s, 72 °C for 45 s, and final incubation at 72 °C for 10 min. After amplification, PCR products were purified by gel extraction and then were quantified by Qubit Fluorometer. The RNA-seq library was prepared using NEBNext Ultra II DNA Library Prep kit following manufacturer’s instruction. The libraries were multiplexed and subjected to MiSeq V3 2 × 301 bp sequencing.

Raw sequences were processed using the pRESTO (version 0.5.13) toolkit^67^. Briefly, the paired-ends MiSeq data was firstly assembled into a full-length Ig sequences, followed by removing the low-quality reads, annotating Ig isotype (IgD, IgM, and IgG), masking the primer regions and yielding the final sequences comprises unique sequence with at least two representative reads. The IMGT/High database of mouse Ig repertoire was used as reference to perform V(D)J alignment using IgBLAST in Change-O (version 0.4.6) tool^68^. The V segment genotypes were inferred using TIgGER^68^. Ig sequences were assigned into clonally related lineages and the full germline sequences were built after preforming automated detection of the clonal assignment threshold. The CDR3 hydrophobicity scale was calculated per reference^69^. Mutations were defined as nucleotides that were different from the inferred germline sequence. The clonal diversity of the repertoire was analyzed using the general form of the diversity index, as proposed by Hill^70^ and implemented in the Alakazam (version 0.3.0)^68^. To determine the selection strength of the CDR and FWR regions the BASELINe tool^71^ was used. The somatic hypermutation targeting models were computed by the SHazaM software (version 0.2.1)^68^. The raw data has been deposited in Gene Expression Omnibus (GSE139225).

### Statistical analysis

The statistical analysis of T- and B-cell responses and nAb titer and B-cell profiling were performed using two-sided Mann–Whitney test in GraphPad Prism 7.0 software (GraphPad Software Inc.). The statistical comparisons between strategies at given immunization on repertories mutation frequency and amino acid features were calculated using unpaired two-sided Wilcoxon test in R.

### Reporting summary

Further information on experimental design is available in the Nature Research Reporting Summary linked to this article.

## Supplementary information

Supplementary Information

Reporting Summary

## Data Availability

The raw sequence data were deposited in Gene Expression Omnibus, accession number: GSE139225. All data used that support the findings of this study are available from the corresponding author upon reasonable request.
