# Supplementary material for: Sequential immunization induces strong and broad immunity against all four dengue virus serotypes
Source: NPJ Vaccines. 2020 Jul 24;5:68. doi: 10.1038/s41541-020-00216-0 (PMC7382454; doi:10.1038/s41541-020-00216-0)
Supplement: Supplementary file 1 — Supplementary Information [file 41541_2020_216_MOESM1_ESM.pdf]

Supplementary Information For

Sequential immunization induces strong and broad immunity against all four dengue virus serotypes

Jue Hou[1], Shubham Shrivastava[1], Hooi Linn Loo[1], Lan Hiong Wong[1], Eng Eong Ooi[1, 2], Jianzhu Chen[1, 3, *]

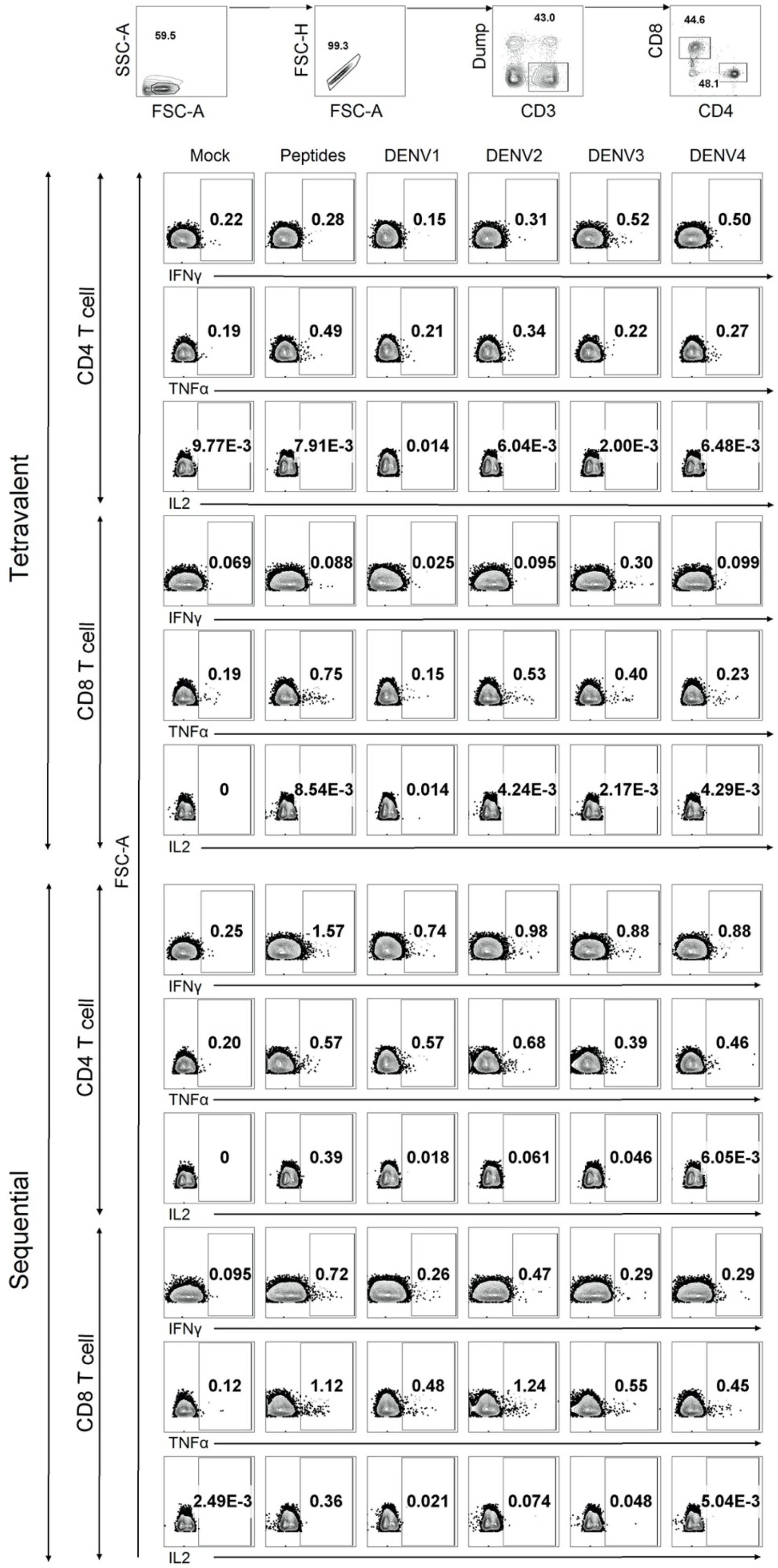
SSC-A
FSC-A
59.5
FSC-H
FSC-A
99.3
Dump
CD3
43.0
CD8
CD4
44.6
48.1
Mock
Peptides
DENV1
DENV2
DENV3
DENV4
Tetravalent
Sequential
CD4 T cell
CD8 T cell
FSC-A
IFNγ
TNFα
IL2
0.22
0.28
0.15
0.31
0.52
0.50
0.19
0.49
0.21
0.34
0.22
0.27
9.77E-3
7.91E-3
0.014
6.04E-3
2.00E-3
6.48E-3
0.069
0.088
0.025
0.095
0.30
0.099
0.19
0.75
0.15
0.53
0.40
0.23
0
8.54E-3
0.014
4.24E-3
2.17E-3
4.29E-3
0.25
1.57
0.74
0.98
0.88
0.88
0.20
0.57
0.57
0.68
0.39
0.46
0
0.39
0.018
0.061
0.046
6.05E-3
0.095
0.72
0.26
0.47
0.29
0.29
0.12
1.12
0.48
1.24
0.55
0.45
2.49E-3
0.36
0.021
0.074
0.048
5.04E-3

Supplementary Figure 1. Flow cytometry gating strategies for T cells in the spleen and the representative profiles of intracellular cytokines staining.

Splenocytes from immunized mice were stimulated with peptides or virus for 6 hours, followed by surface staining for CD3, CD4, CD8, and Dump (F4/80, CD11c, Ly6G, and NK1.1), followed by intracellular staining for IFNγ, TNFα and IL-2. Lymphocytes were gated based FSC vs SSC and used for CD3 vs Dump plots. CD4 vs CD8 staining was shown in $CD3^+$ cells. Expression of intracellular IFNγ, TNFα and IL-2 are shown in $CD4^+$ and $CD8^+$ T cells. The gates for positive intracellular cytokine staining were based on the corresponding non-stimulated controls (mock) and FMO (Fluorescence minus One) controls.

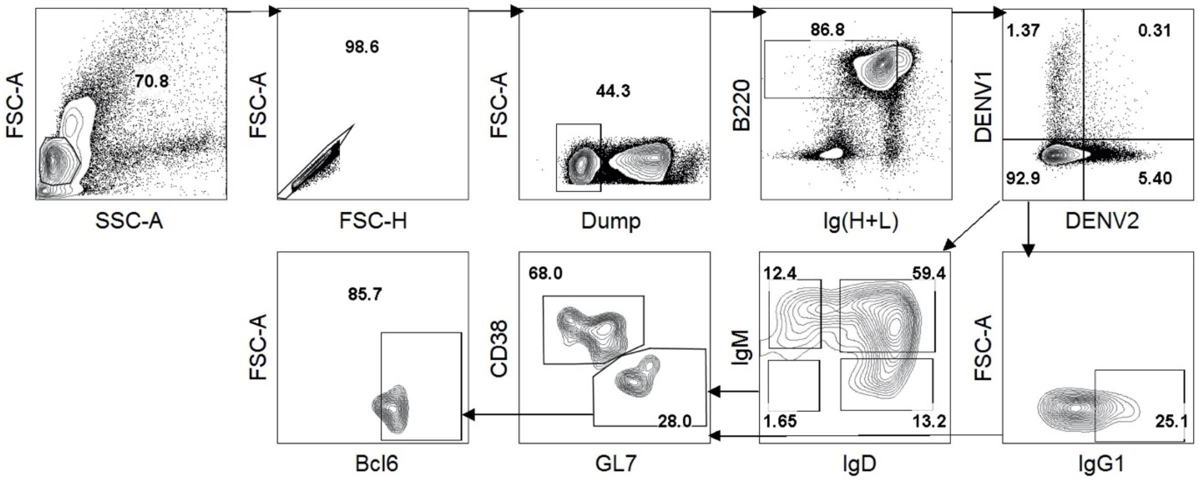

Supplementary Figure 2. Flow cytometry gating strategies for B cells.

Splenocytes from immunized mice were stained for DENV1 and DENV2 specific probes, followed by surface staining for dumps (CD90.2, F4/80, CD11c, CD4, CD8, Ly6G, and NK1.1), B220, Ig $_{(H+L)}$, IgM, IgD, IgG1, GL7, and CD38. B cells were identified by surface B220^{+} and intracellular Ig$_{(H+L)}$ expression. The antigen specific B cells were identified as DENV1^{+}, DENV2^{+} and DENV1^{+}DENV2^{+}. The subsets were distinguished by IgG1, IgD and IgM expression into IgG1^{+}, IgM^{-}IgD^{-}, IgM^{+}IgD^{-}, IgM^{+}IgD^{+}, and IgM^{-}IgD^{+}. In total antigen-specific B cells or each Ig subset, the memory cells (CD38^{+}GL7^{-}) and germinal center cells (CD38^{-}GL7^{+}) were gated for analyzing of intranuclear Bcl-6 expression.

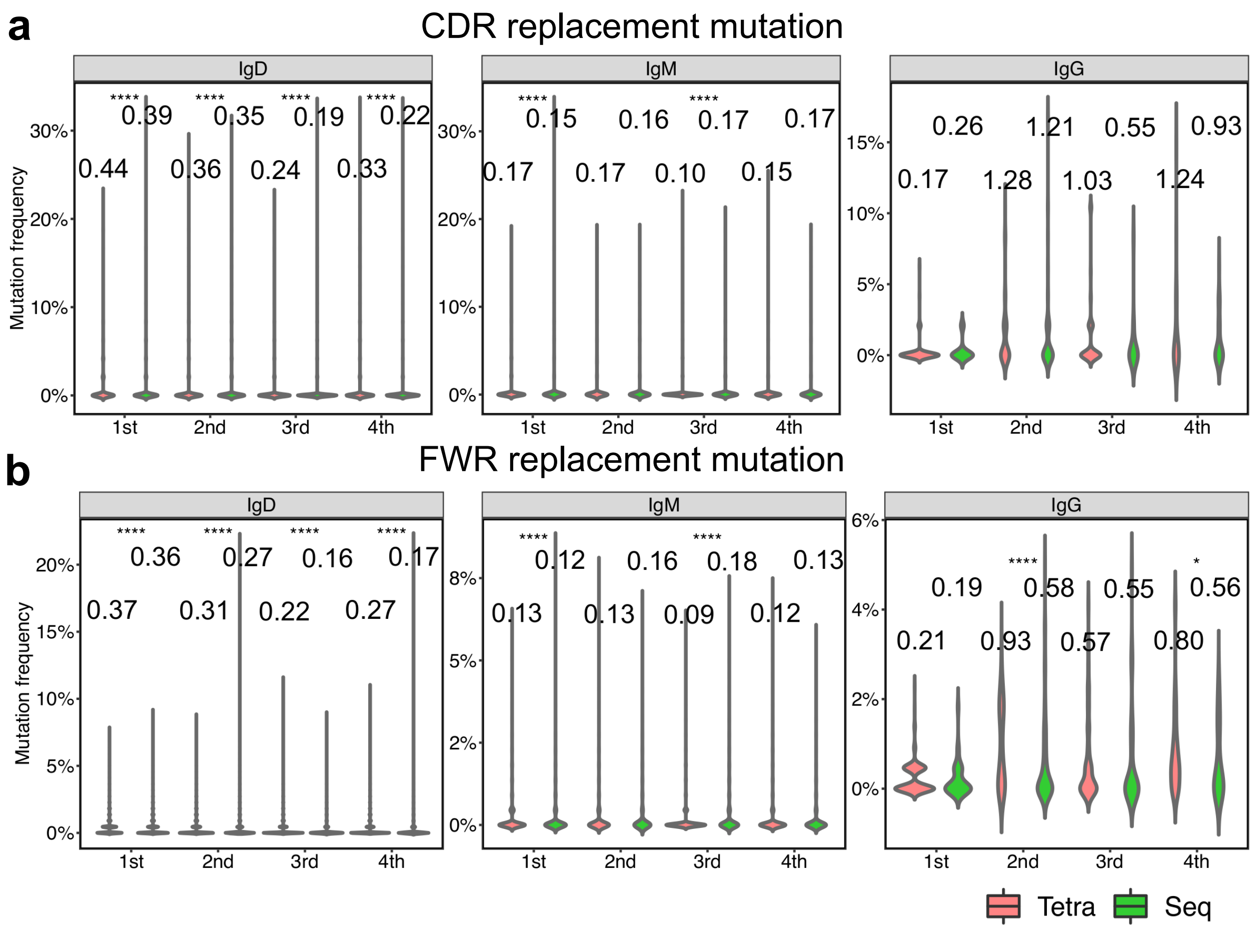

Supplementary Figure 3. The replacement mutation frequencies in CDR and FWR of IgD, IgM and IgG isotypes.

The replacement mutation frequencies in CDR (a) and FWR (b) of IgD, IgM and IgG were calculated and shown as violin plot to present the distribution shape of the data. The value above each violin plot is the mean value of mutation frequency of given group and dose. The labelled asterisk shows the statistical significance by Wilcoxon test analysis between two immunization strategies at a given dose. * $p < 0.05$, ** $p < 0.01$, *** $p < 0.001$, **** $p < 0.0001$.

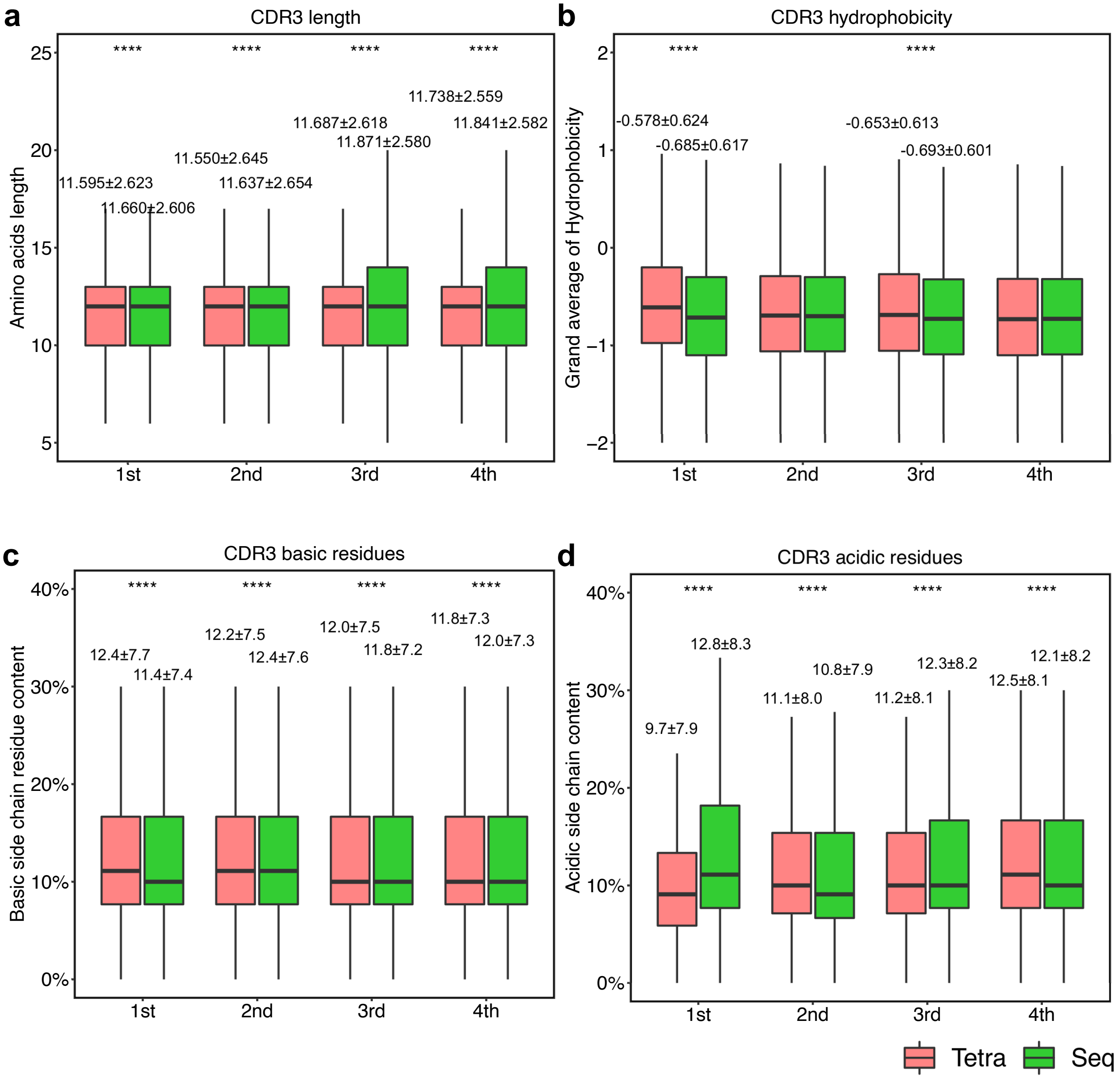

Supplementary Figure 4. The comparisons of CDR3 features between tetravalent and sequential immunizations.

The final Ig repertoires (sequence quantity of each sample referred to Table 2) were used for these analyses. The CDR3 length (a), hydrophobicity (b), percentages of basic residues (c) and acidic residues (d) in CDR3 are shown as mean ± SD. * $p < 0.05$, ** $p < 0.01$, *** $p < 0.001$, **** $p < 0.0001$ by Wilcoxon test analysis.

Supplementary Table 1 Flow cytometry antibody list

Assay	Color	Mab	Clone	Isotype	Catalog	Company	Conc. (mg/ml)	Work Volume (μL)
T cell	APC-Cy7	FVS780	Viability	---	565388	BD	---	0.25
	APC-Cy7	F4/80	BM8	Rat, IgG2a, κ	123118	Biolegend	0.2	0.75
	APC-Cy7	CD11c	N418	Ar Ham, IgG, κ	117324	Biolegend	0.2	0.25
	APC-Cy7	Ly-6G (Gr-1)	RB6-8C5	Rat, IgG2b, κ	557661	BD	0.2	0.5
	APC-Cy7	NK1.1	PK136	Ms, IgG2a, κ	560618	BD	0.2	0.5
	BUV395	CD3e	145-2C11	Ar Ham IgG1, κ	563565	BD	0.2	1.5
	AF647	CD4	RM4-5	Rat IgG2a, κ	557681	BD	0.2	1
	PerCP-Cy5.5	CD8a	53-6.7	Rat IgG2a, κ	551162	BD	0.2	1
	BUV737	IFNγ	XMG1.2	Rat IgG1, κ	564693	BD	0.2	2
	PE	TNFα	MP6-XT22	Rat IgG1	554419	BD	0.2	2
	PE-Cy5	IL2	JES6-5H4	Rat IgG2b, κ	503824	Biolegend	0.2	1
B cell and sorting	APC-Cy7	FVS780	Viability	---	565388	BD	---	0.25
	APC-Cy7	CD90.2 (Thy-1.2)	53-2.1	Rat, IgG2a, κ	561641	BD	0.2	0.25
	APC-Cy7	F4/80	BM8	Rat, IgG2a, κ	123118	Biolegend	0.2	0.75
	APC-Cy7	CD11c	N418	Ar Ham, IgG, κ	117324	Biolegend	0.2	0.25
	APC-Cy7	CD4	GK1.5	Rat, IgG2b, κ	552051	BD	0.2	0.25
	APC-Cy7	CD8a	53-6.7	Rat, IgG2b, κ	557654	BD	0.2	1
	APC-Cy7	Ly-6G (Gr-1)	RB6-8C5	Rat, IgG2b, κ	557661	BD	0.2	0.5
	APC-Cy7	NK1.1	PK136	Ms, IgG2a, κ	560618	BD	0.2	0.5
	AF546	Den1	---	---	---	In-house	---	0.5
	AF647	Den2	---	---	---	In-house	---	1
	PE-CF594	Bcl-6	K112-91	mouse IgG1, κ	562401	BD	---	5
	PE-Cy7	IgM	R6-60.2	Rat, IgG2a, κ	552867	BD	0.2	0.3
	PerCP-Cy5.5	IgD	11-26c	Rat, IgG2a, κ	564273	BD	0.2	0.3
	FITC	GL7	GL7	Rat, IgM, κ	553666	BD	0.5	1
	BUV737	CD45R (B220)	RA3-6B2	Rat, IgG2a, κ	564449	BD	0.2	0.3
	BUV395	CD38	90	Rat, IgG2a, κ	740245	BD	0.2	0.3
	AF 350	IgG (H+L) F(ab)'2	---	Goat	A-11068	Life Tech	2	0.3
		IgG1-Biotin	A85-1	Rat, IgG1, κ	550331	BD	0.5	1
	BV786	Streptavidin	---	---	563858	BD	0.1	0.3

Supplementary Table 2 The pre-amplification PCR Primers for germinal center B cell genes

Sequence Name	Sequence (5’-3’)
Irf3-For	GAGAGCCGAACGAGGTTCA
Irf3-Rev	AGCAGCTAACCGCAACACTT
Irf4-For	CAGGAGCTGGAGGGATTATG
Irf4-Rev	GGCTTCAGCAGACCTTATGC
Irf5-For	CAGTACCCAGGGCTTCAGTG
Irf5-Rev	GCTTTTGTTAAGGGCACAGC
Irf8-For	GATCGAACAGATCGACAGCA
Irf8-Rev	TCTTCAAAATCTGGGCTCTTG
Ifnar1-For	CCCAGAGTTCACCCTCAAGA
Ifnar1-Rev	TGAGTGGTGGGAAGCACAC
Ifnar2-For	GGAGGGCATGGAGAGCTAC
Ifnar2-Rev	TTTTGGAAGTGACAGGTGGA
Ifngr1-For	GTGCCTGTACCGACGAATG
Ifngr1-Rev	TTGACCGTGCATAGTCAGATTC
Nfkb1-For	AGGCCATTGAAGTGATCCAG
Nfkb1-Rev	CAGAGATAGCAGTGGGCTGTC
Nfkb2-For	AGCGTTGCTGGACTACGG
Nfkb2-Rev	GTGGTTGGTGAGGTTGATGA
Nfatc1-For	GGTGCTGTCTGGCCATAACT
Nfatc1-Rev	GAAACGCTGGTACTGGCTTC
Mki67-For	CGTGTATCCTTTGGTGGACA
Mki67-Rev	CACTGGAAGTCCTGCCTGAT
Bcl6-For	AGTTTACCCGGCACGCTAGT
Bcl6-Rev	TATTGCCTTCCCTCAGGTTG
Myc-For	TCCTGTACCTCGTCCGATTC
Myc-Rev	GCACCTCTTGAGGACCAGTG
Tbx21-For	CGCGAGGACTACGCATTG
Tbx21-Rev	CTTTCCACACTGCACCCACT
Slamf1-For	GTCTGCGATTGCTGGCTAA
Slamf1-Rev	GAGGATGCGGACACTTTTGT
Tnfrsf13c-For	GGACTGATACTGGCGCTGAC
Tnfrsf13c-Rev	CGCTGTCTGCATCTTCTTTG
Prdm1-For	ACAGAGGCCGAGTTTGAAGA
Prdm1-Rev	GGCATTCTTGGGAACTGTGT
Fas-For	CGCTGTTTTCCCTTGCTG
Fas-Rev	CAAAATGGGCCTCCTTGATA
Polh-For	GCTACTCGGGAGCAGGTACA

Polh-Rev	GCTCATCTTGTGAGCGTCAT
Cd40-For	GTGACTTGTGGCTTCAGCAG
Cd40-Rev	CTGTGCAGTGGCTTGTCAGT
Cd69-For	GAAGATGCTGCTACTCTTGCTG
Cd69-Rev	TGGTGCTATGGCACAGTCAT
Cd79a-For	ACGATGCCAGGGGGTCTA
Cd79a -Rev	ACTGAAGGCTGAACCACCAT
Cd79b-For	CACACTGGTGCTGTCTTCCA
Cd79b-Rev	TGCACCTGAGTGGTTTGTGT
Cd80-For	CCCGAGTATAAGAACCGGACT
Cd80-Rev	TCCATTTTCCAACCAAGAGAA
Cd83-For	ACCGTGGTTCTGAAGGTGAC
Cd83-Rev	ATGTGCCCTTGGCTTTGTAA
Cd86-For	GCTGAGGAAGAAAGAGGAGCA
Cd86-Rev	CCAGCTCACTCAGGCTTATG
Cxcr4-For	AATTTTGTTGCCTGGTGCAG
Cxcr4-Rev	GGTGGGCAGGAAGATCCTAT
Actb-For	CTGTATTCCCCTCCACGTG
Actb-Rev	CCATCACAATGCCTGTGGTA

Supplementary Table 3 The TaqMan probes for qPCR assay

Gene Symbol	Alias	Reference Sequence ID	External Forward 5'-3'	External Reverse 5'-3'	TaqMan Assay ID
Irf3	Irf3	NM_016849.4	GAGAGCCGAACGAGGTTCA	AGCAGCTAACCGCAACACTT	Mm01203177_m1
Irf4	Irf4	NM_013674.1	CAGGAGCTGGAGGGATTATG	GGCTTCAGCAGACCTTATGC	Mm00516431_m1
Irf5	Irf5	NM_012057.4	CAGTACCCAGGGCTTCAGTG	GCTTTTGTTAAGGGCACAGC	Mm00496477_m1
Irf8	Irf8	NM_008320.3	GATCGAACAGATCGACAGCA	TCTTCAAAATCTGGGCTCTTG	Mm00492567_m1
Ifnar1	IFNaR1	NM_010508.2	CCCAGAGTTCACCCTCAAGA	TGAGTGGTGGGAAGCACAC	Mm00439544_m1
Ifnar2	IFNaR2	NM_001110498.1	GGAGGGCATGGAGAGCTAC	TTTTGGAAGTGACAGGTGGA	Mm00494916_m1
Ifngr1	IFNgR1	NM_010511.2	GTGCCTGTACCGACGAATG	TTGACCGTGCATAGTCAGATTC	Mm00599890_m1
Nfkb1	NFkB1	NM_008689.2	AGGCCATTGAAGTGATCCAG	CAGAGATAGCAGTGGGCTGTC	Mm00476379_m1
Nfkb2	NFkB2	NM_001177369.1	AGCGTTGCTGGACTACGG	GTGGTTGGTGAGGTTGATGA	Mm00479807_m1
Nfatc1	NFATc1	NM_001164109.1	GGTGCTGTCTGGCCATAACT	GAAACGCTGGTACTGGCTTC	Mm00479445_m1
Mki67	Ki67	NM_001081117.2	CGTGTATCCTTTGGTGGACA	CACTGGAAGTCCTGCCTGAT	Mm01278617_m1
Bcl6	Bcl6	NM_009744.3	AGTTTACCCGGCACGCTAGT	TATTGCCTTCCCTCAGGTTG	Mm00477633_m1
Myc	cMyc	NM_010849.4	TCCTGTACCTCGTCCGATTC	GCACCTCTTGAGGACCAGTG	Mm00487804_m1
Tbx21	Tbet	NM_019507.2	CGCGAGGACTACGCATTG	CTTTCCACACTGCACCCACT	Mm00450960_m1
Slamf1	SLAM	NM_013730.4	GTCTGCGATTGCTGGCTAA	GAGGATGCGGACACTTTTGT	Mm00443316_m1
Tnfrsf13c	BAFFR	NM_028075.2	GGACTGATACTGGCGCTGAC	CGCTGTCTGCATCTTCTTTG	Mm00840578_g1
Prdm1	Blimp1	NM_007548.3	ACAGAGGCCGAGTTTGAAGA	GGCATTCTTGGGAACTGTGT	Mm00476128_m1
Fas	Fas	NM_007987.2	CGCTGTTTTCCCTTGCTG	CAAAATGGGCCTCCTTGATA	Mm00433237_m1
Polh	Poln	NM_030715.3	GCTACTCGGGAGCAGGTACA	GCTCATCTTGTGAGCGTCAT	Mm00453168_m1
Cd40	CD40	NM_170702.2	GTGACTTGTGGCTTCAGCAG	CTGTGCAGTGGCTTGTCAGT	Mm00441891_m1
Cd69	CD69	NM_001033122.3	GAAGATGCTGCTACTCTTGCTG	TGGTGCTATGGCACAGTCAT	Mm01183380_m1
Cd79a	Iga	NM_007655.3	ACGATGCCAGGGGGTCTA	ACTGAAGGCTGAACCACCAT	Mm00432423_m1
Cd79b	Igb	NM_008339.2	CACACTGGTGCTGTCTTCCA	TGCACCTGAGTGGTTTGTGT	Mm00434143_m1
Cd80	CD80	NM_009855.2	CCCGAGTATAAGAACCGGACT	TCCATTTTCCAACCAAGAGAA	Mm00711660_m1
Cd83	CD83	NM_009856.2	ACCGTGGTTCTGAAGGTGAC	ATGTGCCCTTGGCTTTGTAA	Mm00486868_m1

Cd86	CD86	NM_019388.3	GCTGAGGAAGAAAGAGGAGCA	CCAGCTCACTCAGGCTTATG	Mm01344638_m1
Cxcr4	CXCR4	NM_009911.3	AATTTTGTTGCCTGGTGCAG	GGTGGGCAGGAAGATCCTAT	Mm01292123_m1
Actb	Bactin	NM_007393.3	CTGTATTCCCCTCCACGTG	CCATCACAATGCCTGTGGTA	Mm02619580_g1

Supplementary Table 4 The murine immunoglobulin genes RNA-seq PCR primers

Primer	Annotations	Sequences
	First-strand cDNA synthesis	
SmartNNNa	5-template-switch adaptor. U = dU	AAGCAGUGGTAUCAACGCAGAGUNNNNUNNNNUNNNNUCTT(rG)4
	IGH cDNA synthesis primer mix	
mIGG12_r1	cDNA synthesis, mouse IgG1/IgG2 heavy-chain mRNA	KKACAGTCACTGAGCTGCT
mIGG3_r1	cDNA synthesis, mouse IgG3 heavy-chain mRNA	GTACAGTCACCAAGCTGCT
mIGA_r1	cDNA synthesis, mouse IgA heavy-chain mRNA	CCAGGTCACATTCATCGTG
mIGM_r1	cDNA synthesis, mouse IgM heavy-chain mRNA	CTGGATGACTTCAGTGTTGT
mIGD_r1	cDNA synthesis, mouse IgD heavy-chain mRNA	GCCATTTCTCATTTCAGAGG
mIGE_r1	cDNA synthesis, mouse IgE heavy-chain mRNA	GTTCACAGTGCTCATGTTC
	First PCR amplification	
M1SS	Step-out primer 1, anneals on the switch adaptor	AAGCAGTGGTATCAACGCA
	IGH reverse primer mix	
MIGG12_r2	Nested primer with Z adaptor, mouse IgG1/IgG2 heavy-chain cDNA	ATTGGGCAGCCCTGATTAGTGGATAGACMGATG
mIGG3_r2	Nested primer with Z adaptor, mouse IgG3 heavy-chain cDNA	ATTGGGCAGCCCTGATTAAGGGATAGACAGATG
mIGA_r2	Nested primer with Z adaptor, mouse IgA heavy-chain cDNA	ATTGGGCAGCCCTGATTTCAGTGGGTAGATGGTG
mIGM_r2	Nested primer with Z adaptor, mouse IgM heavy-chain cDNA	ATTGGGCAGCCCTGATTGGGGGAAGACATTTGG
mIGD_r2	Nested primer with Z adaptor, mouse IgD heavy-chain cDNA	ATTGGGCAGCCCTGATTCTCTGAGAGGAGGAAC
mIGE_r2	Nested primer with Z adaptor, mouse IgE heavy-chain cDNA	ATTGGGCAGCCCTGATTAAGGGGTAGAGCTGAG
	Second PCR amplification	
	IGH	
VH1	VH1 External 1	agRtYcagctgcaRcagtct
VH1_2	VH1 External 2	aggtccaactgcagcagcc
VH2	VH2 External	tctgcctggtgacWttccca
VH3	VH3 External	gtgcagcttcaggagtcag
VH4	VH4 External	gaggtgaagcttctcgagtc
VH5	VH5 External	gaagtgaagctggtggagtc
VH6	VH6 External	atgKacttgggactgaRctgt
VH7	VH7 External	cagtgtgaggtgaagctggt
VH8	VH8 External	ccaggttactctgaaagagtc
VH9	VH9 External	tgtggaccttgctattcctga

VH10	VH10 External	tgttggggctgaagtgggttt
VH11	VH11 External	atggagtgggaactgagctta
VH12	VH12 External	agcttcaggagtcaggacc
VH13	VH13 External	caggtgcagcttgtagagac
VH14	VH14 External	atgcagctgggtcatcttctt
VH15	VH15 External	gactggatttggatcacKctc
VH16	VH16 External	tggagtttggacttagttggg
Cμ	C mu External	catggccaccagattcttatc
Cγ	C gamma External 1	agggaaataRcccttgaccag
Cγ_2	C gamma External 2	agggaagtagcctttgacaag
Cδ	C delta External	tctgagaggaggaacatgtc
